# Single-molecule tracking (SMT) and localization of SRF and MRTF transcription factors during neuronal stimulation and differentiation

**DOI:** 10.1098/rsob.210383

**Published:** 2022-05-11

**Authors:** Oliver Kuchler, Jule Gerlach, Thomas Vomhof, Johannes Hettich, Julia Steinmetz, J. Christof M. Gebhardt, Jens Michaelis, Bernd Knöll

**Affiliations:** ^1^ Institute of Neurobiochemistry, Ulm University, Albert-Einstein-Allee 11, 89081 Ulm, Germany; ^2^ Institute of Biophysics, Ulm University, Albert-Einstein-Allee 11, 89081 Ulm, Germany; ^3^ Department of Statistics, TU Dortmund University, August-Schmidt Straße 1, 44227 Dortmund, Germany

**Keywords:** SRF, MRTF, snap-tag, halo-tag, single-molecule, transcription

## Abstract

In cells, proteins encoded by the same gene do not all behave uniformly but engage in functional subpopulations induced by spatial or temporal segregation. While conventional microscopy has limitations in revealing such spatial and temporal diversity, single-molecule tracking (SMT) microscopy circumvented this problem and allows for high-resolution imaging and quantification of dynamic single-molecule properties. Particularly in the nucleus, SMT has identified specific DNA residence times of transcription factors (TFs), DNA-bound TF fractions and positions of transcriptional hot-spots upon cell stimulation. By contrast to cell stimulation, SMT has not been employed to follow dynamic TF changes along stages of cell differentiation. Herein, we analysed the serum response factor (SRF), a TF involved in the differentiation of many cell types to study nuclear single-molecule dynamics in neuronal differentiation. Our data in living mouse hippocampal neurons show dynamic changes in SRF DNA residence time and SRF DNA-bound fraction between the stages of adhesion, neurite growth and neurite differentiation in axon and dendrites. Using TALM (tracking and localization microscopy), we identified nuclear positions of SRF clusters and observed changes in their numbers and size during differentiation. Furthermore, we show that the SRF cofactor MRTF-A (myocardin-related TF or MKL1) responds to cell activation by enhancing the long-bound DNA fraction. Finally, a first SMT colocalization study of two proteins was performed in living cells showing enhanced SRF/MRTF-A colocalization upon stimulation. In summary, SMT revealed modulation of dynamic TF properties during cell stimulation and differentiation.

## Graphical abstract

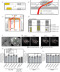

## 1. Introduction

After cell cycle exit, cells undergo a set of differentiation processes including substrate adhesion, contact formation, cell growth and morphological alterations. The latter include changes of cell shape (e.g. myocytes adopting spindle shapes). In neurons, differentiation involves enormous growth of neurites later on differentiating into axons (typically one) and several dendrites [1]. This process of neuronal polarization is divided in several stages: stage 1 is adhesion, stage 2 is growth of 4–5 neurites, stage 3 is rapid elongation of one neurite (the future axon) and stage 4 is differentiation of the shorter neurites into dendrites [1].

From a molecular perspective, the process of cell differentiation has so far mainly been analysed by bulk analysis of all molecules in a given cell. Thus, quantitative parameters were achieved by averaging over all molecules implicitly assuming that the entire molecule population behaves homogeneously. In recent years, single-molecule tracking (SMT; also called single-molecule localization microscopy; SMLM) has allowed for analysing single molecules thereby identifying distinct properties of molecule subpopulations in a cell. SMT has been particularly successful in the nucleus to identify dynamic properties of chromatin-associated molecules such as transcription factors [2,3]. So far, several parameters of TF nuclear kinetics have been determined, including exact DNA binding times (ranging from a few seconds to 1–2 min), the TF fraction bound to DNA and localizations of transcriptional ‘hot-spots’ [4–11]. Previous studies have analysed the impact of cell stimulation by, for example, growth factor, hormone administration or DNA damage on TF-DNA interaction, and demonstrated that DNA occupancy and bound fraction of TFs such as p53, SRF, GR, ER, CREB and SOX2 are enhanced by cell activation [4–10].

By contrast, so far, to the best of our knowledge no SMT study has addressed such dynamic changes in TF parameters during cell differentiation for any cell type. However, employing other techniques such as fluorescence correlation spectroscopy (FCS) showed distinct changes in Oct4 and Sox2 dynamics in embryonic cell differentiation [12,13]. In this study, we provide such a first SMT analysis focusing on several stages of neuronal differentiation of mouse hippocampal neurons.

We focus on SRF (serum response factor), a TF almost ubiquitously expressed in all cell types [14–17]. In a previous study we demonstrated that DNA residence time and DNA bound-fraction of SRF is enhanced by cell stimulation with serum or growth factors such as BDNF (brain-derived neurotrophic factor; [5]). By contrast to SRF, its partner proteins of the MRTF (myocardin-related TFs) family (MRTF-A and MRTF-B also known as MKL1 and MKL2; [17–20]) have not been yet investigated with SMT up until now. SRF/MRTFs form a gene regulatory complex involved in regulation of genes encoding for actin cytoskeletal genes and immediate early genes (IEGs) such as *cFos, Egr1, Npas4* and *Arc* [17–20]. Notably, SRF/MRTF activity not only regulates genes encoding for the actin cytoskeleton (*Actb, Actc, tropomyosin, vinculin*) but has the unique property of being adjusted by cytosolic and nuclear actin dynamics. Thus, monomeric G-actin inhibits whereas polymerized F-actin enhances SRF/MRTF activity [17–20].

Mouse mutagenesis of either S*rf* or *Mrtfa/b* compound mutants has shown defects in cellular differentiation in many cell types including cardiomyocytes, myocytes, hepatocytes and keratinocytes [17–20]. In neurons, SRF and MRTFs modulate several differentiation processes including migration, neurite protrusion, axonal and dendritic differentiation as well as synapse function [21–28]. Given that the SRF/MRTF complex is widely involved in cell differentiation we determined several dynamic parameters for both TFs including a colocalization study of both TFs in living cells. For this, SRF and MRTF-A were fused with tags allowing for SMT in particular the HALO-tag derived from the bacterial haloalkane dehalogenase enzyme and the SNAP-tag derived from the DNA repair protein O6-alkylguanine-DNA alkyltransferase [2,29,30]. For both tags reagents with photo-stable organic fluorophores such as TMR (Tetramethylrhodamine), SiR (Silicon Rhodamine-like) or Janelia dyes exist ideally suited for SMT [2,29,30].

In this study, we showed that SRF residence time and DNA-bound fraction were altered in neurons while passaging through several stages of differentiation. This was accompanied by changes in number and size of SRF foci or clusters during neuronal polarization. Furthermore, we provide first dynamic SMT properties of MRTF-A and demonstrate that MRTF-A DNA binding properties were enhanced by cell stimulation. Finally, a SMT colocalization study of SRF and MTRF-A in living fibroblasts supports models of enhanced SRF/MRTF-A complex formation by cell activation.

## 2. Material and methods

### 2.1. Cloning and lentivirus production

We fused sequences encoding for the Halo-Tag to the N-terminus of the wild-type mouse *Srf* sequence (UniProtKB Q9JM73) and the SNAP-Tag to the N-terminus of the wild-type mouse MRTF-A sequence (UniProtKB Q8K4J6) and cloned them into a lentiviral expression vector as previously described [5]. Together with pMd2.g (addgene #12259) and psPax2 (addgene #12260), pLV-TetO-vectors of the desired constructs were transiently transfected in LentiX-293T cells. The SRF *α*I helix construct was previously described [5,31]. The generated lentiviruses were harvested and stored at −80°C until further use.

### 2.2. Cell culture

#### 2.2.1. Primary mouse hippocampal neurons

**Figure 1.**
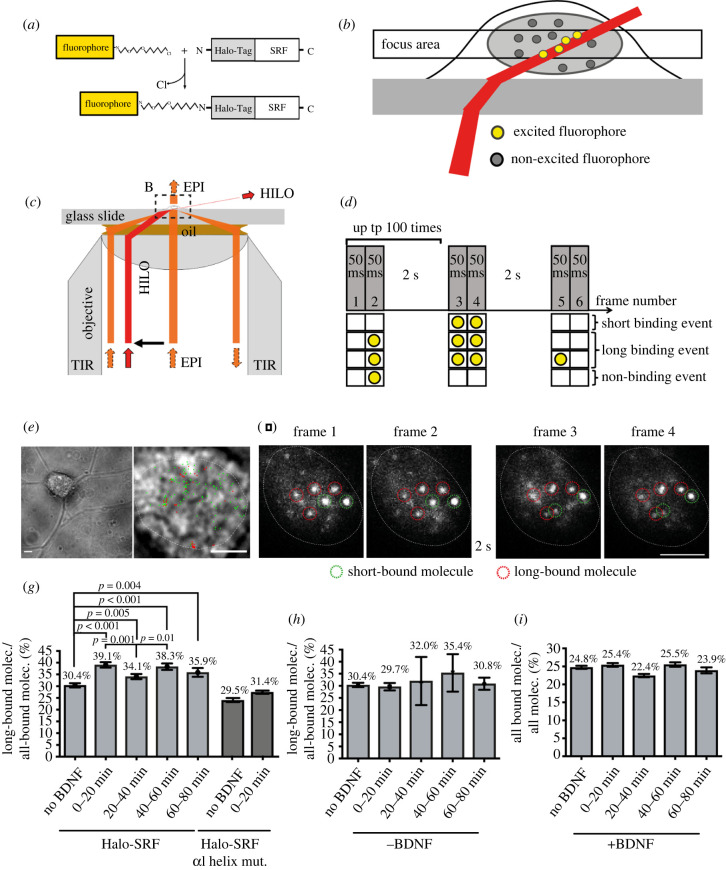
Neuronal activation with BDNF enhances the long-bound DNA fraction of SRF. (*a*) Schematic showing fluorescent labelling of SRF molecules using a Halo-tag and a Halo-Tag specific Janelia Fluor 646 (JF646) fluorophore. (*b*) Schematic illustrating the excitation scheme for live-cell imaging of Halo-SRF using HILO illumination. (*c*) Schematic of the beampath within the microscope objective for HILO illumination in comparison to epi and TIR illumination. (*d*) Scheme of interlaced time-lapse microscopy (ITM). Here, two consecutive frames of 50 ms illumination were followed by a 2 s dark time for 100 repeats. Molecules detected in two consecutive illumination frames were short binding events. A molecule surviving at least one dark-time was counted as a long binding event. Molecules visible in only one illumination frame were considered non-binding molecules. (*e*) Exemplary frame from a ITM movie of a neuron lentivirally transduced with Halo-SRF overlayed with the determined position from all bound molecules for the respective movie (short binding (green) and long-bound (red) molecules). Superimposed is the outline of the cell nucleus (white dashed line) determined from the respective bright field images. (*f*) Representative examples of four consecutive frames from an ITM movie. Long-bound molecule were present in all four frames (red dashed circles). Short-bound molecules were present in two consecutive frames only (green dashed circles). (*g*) Neurons expressing Halo-SRF (light grey bars) or mutated SRF *α*I helix (dark grey bars) were stimulated for indicated time intervals with BDNF and the percentage of long-bound molecules versus all bound molecules was determined from ITM movies. *n* = 2472 molecules, 60 cells (no BDNF); *n* = 1782 molecules, 45 cells (0–20 min BDNF); *n* = 1807 molecules, 38 cells (20–40 min BDNF); *n* = 1224 molecules, 26 cells (40–60 min BDNF); *n* = 655 molecules, 13 cells (60–80 min BDNF). *n* (SRF *α*I helix) = 1889 molecules, 21 cells (no BDNF); *n* (SRF *α*I helix) = 2859 molecules, 33 cells (0–20 min BDNF). (*h*) Fraction of long-bound Halo-SRF events versus all binding events determined by ITM from neurons during 80 min without BDNF. *n* = 2472 molecules (no BDNF); *n* = 1202 molecules (0–20 min no BDNF); *n* = 1749 molecules (20–40 min no BDNF); *n* = 1043 molecules (40–60 min no BDNF); *n* = 505 molecules (60–80 min no BDNF). (*i*) Fraction of all bound SRF molecules determined from ITM movies with and without BDNF stimulation. *n* = 9986 molecules (no BDNF); *n* = 7013 molecules (0–20 min BDNF); *n* = 8050 molecules (20–40 min BDNF); *n* = 4798 molecules (40–60 min BDNF); *n* = 2739 molecules (60–80 min BDNF). Data in (*h-i*) depict molecule-wise analysis with mean ± error. *p*-values and errors were calculated by two-sample binomial test. Scale bars: (*e*) 10 µm; (*e*, zoom) 5 µm; (*f*) 5 µm.

**Figure 2.**
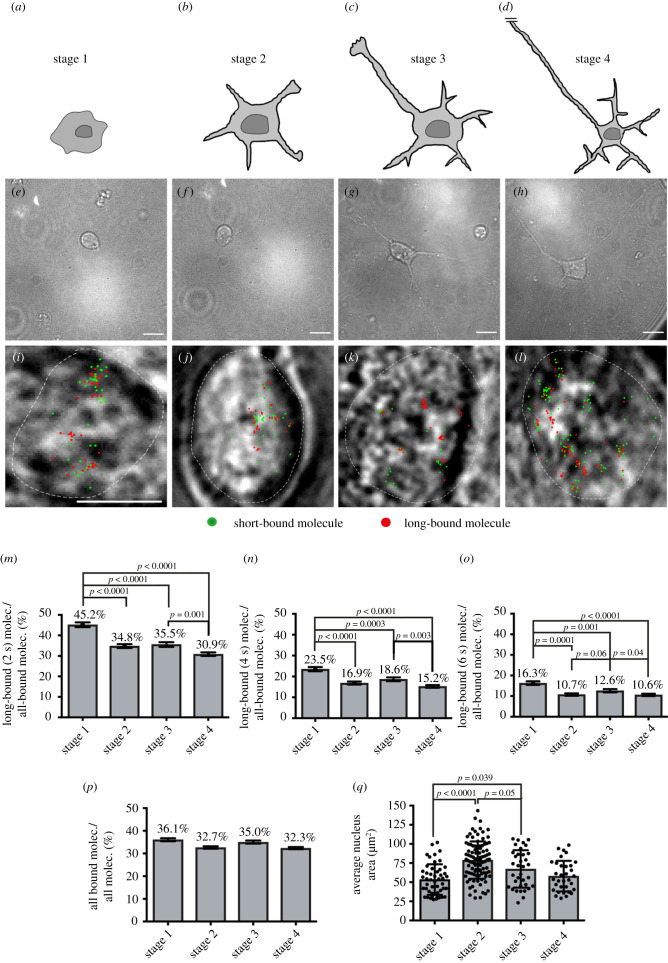
The long-bound SRF fraction is enhanced at the initial stage of neuronal polarization. (*a–d*) Schematics illustrating stages of neuronal differentiation, namely initial cell adhesion (stage 1, 5 h after plating), neurite formation (stage 2, 24 h), axon specification (stage 3, 72 h) and axon-dendrite maturation and contact formation (stage 4, 7d). (*i–l*) Exemplary frames from ITM movies overlayed with the determined position from all bound molecules for the respective movie (short binding (green) and long-bound (red) molecules). Superimposed is the outline of the cell nucleus (white dashed line) determined from the respective bright field images. (*i–l*) Bright-field captures of nuclei (white dashed line) overlayed with a merged picture of the ITM movie indicating short- (green) and long-bound (red) molecules. (*m–o*) Fraction of long-bound versus all bound Halo-SRF molecules for the four differentiation stages determined from ITM movies using 2 s (*m*), 4 s (*n*) or 6 s (*o*) dark-times. (*n* = 1747 molecules in stage 1; *n* = 2465 molecules in stage 2; *n* = 1771 molecules in stage 3; *n* = 4827 molecules in stage 4). (*p*) Halo-SRF bound fraction (short and long binding) determined using ITM movies for all four differentiation stages. The fraction of all-bound Halo-SRF molecules was highest in stage 1, however no statistical significance (*n* = 4850 molecules, 49 cells in stage 1; *n* = 7506 molecules, 98 cells in stage 2; *n* = 5002 molecules, 35 cells in stage 3; *n* = 15 241 molecules, 33 cells in stage 4). Data represent molecule-wise analysis with mean ± error. *p* values and errors were calculated by the two-sample binomial test. (*q*) The average nucleus area/neuron was measured from the bright field image for each of the four differentiation stages. In stage 1, nucleus area was the lowest and in stage 2 the highest area was measured. Each dot represents one cell. Data are depicted as mean ± s.d. *p*-values were calculated by a two-sided ANOVA test. Scale bars: (*e–h*) 10 µm; (*i–l*) 5 µm.

**Figure 3.**
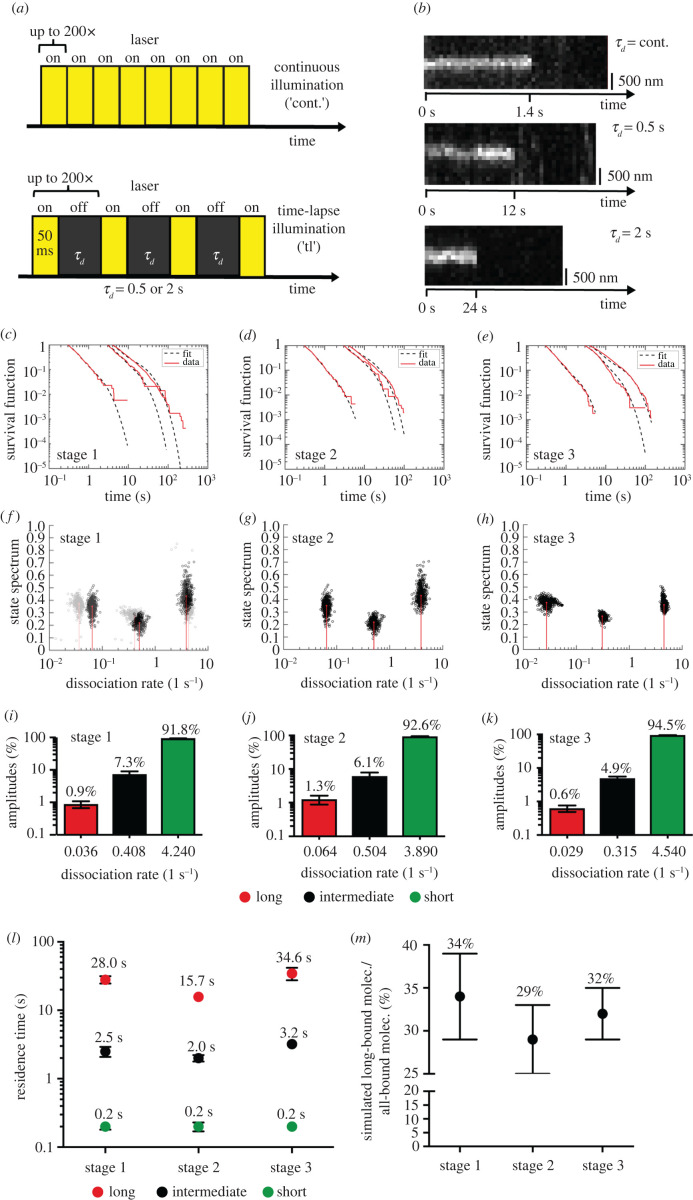
DNA residence times change during neuronal polarization. (*a*) Time-lapse illumination scheme for measuring residence time. The dark-time between two 50 ms illumination frames was varied between 0 (continuous movies) to 2 s. (*b*) Representative kymographs of bound-molecules under different time-lapse conditions. *y*-axes depict position and *x*-axes indicate time. (*c–e*) Computed probabilities of molecules binding for longer than a certain binding time computed from tracked Halo-SRF molecules using different time-lapse conditions (cont., 0.5 s and 2 s), shown are actual data (red) and fitted survival time functions obtained by a three-component decay model (black dashed line) for neurons in stage 1 (*c*), stage 2 (*d*) and stage 3 (*e*). (*f–h*) State spectra of Halo-SRF molecules obtained by a three-component decay model using all data (red lines) and a superposition of 500 results obtained by resampling of 80% of the data (black circles) as an error estimation for neurons in differentiation stage 1 (*f*), stage 2 (*g*) and stage 3 (*h*). (*i–k*) Event spectrum of the corresponding dissociation rates of Halo-SRF. The event spectra show the percentage distribution of Halo-SRF molecules for each calculated dissociation rate for neurons in stage 1 (*i*), stage 2 (*j*) and stage 3 (*k*). Data are presented as mean ± error resulting from three-component decay model fit. (*l*) Average DNA residence times obtained from dissociation rates for short-(green), intermediate (black) and long-bound (red) Halo-SRF molecules. Data were presented as mean ± s.d. $\tau_{tl}=\mathrm{cont}.$: *n* = 387 molecules (stage 1), *n* = 530 molecules (stage 2), *n* = 555 molecules (stage 3); $\tau_{tl}=500\;\mathrm{ms}$: *n* = 343 molecules (stage 1), *n* = 314 molecules (stage 2), *n* = 525 molecules (stage 3); $\tau_{tl}=2\;s$: *n* = 2400 molecules (stage 1), *n* = 1037 molecules (stage 2), *n* = 864 molecules (stage 3). (*m*) Simulated long-bound fraction of the Halo-SRF molecules were simulated from the time-lapse dataset which was used to compute the DNA residence times. For long-bound fraction simulation, an ITM spectrum was calculated, which is corrected for the fact that short binding events are not detected during the dark-time in ITM (see Material and methods). This ITM spectrum was used to simulate the long-bound fraction. Here long-bound and short-bound events were classified by applying the same rules like in the evaluation of measured ITM movies. The results indicate an elevation of the Halo-SRF long-bound fraction in stage 1 neurons compared to those in differentiation stage 2 or 3. Data are presented as mean ± s.d.

**Figure 4.**
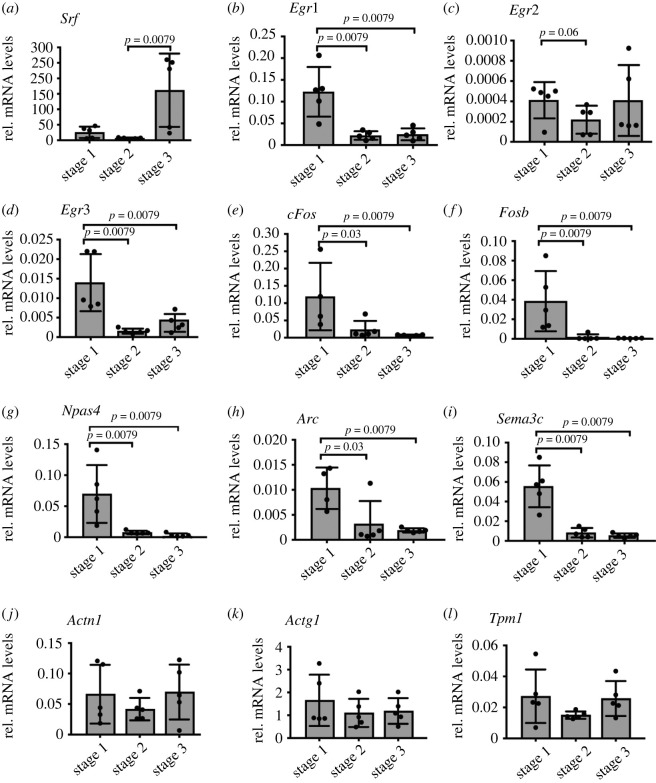
Immediate early gene mRNA abundance but not that of actin cytoskeletal genes changes during neuronal differentiation. Halo-SRF expressing neurons at all three stages were subjected to qPCR analysis for genes indicated. (*a*) Relative mRNA expression levels of *Srf* indicate SRF overexpression in all stages but strongest in stage 3. (*b–h*) All tested SRF-regulated IEGs (as indicated) had the highest mRNA abundance in stage 1 compared to stages 2 and 3. (*i*) Relative mRNA expression level of Sema3c, an SRF-regulated axon guidance gene was increased in stage 1 compared to stage 2 and 3. (*j–l*) mRNA abundance of the tested SRF-regulated actin cytoskeletal genes (as indicated) did not result in significant changes between the differentiation stages. All data are displayed as mean ± s.d. Each dot in (*a–l*) was one independent culture (*n* = 5). *p*-values were calculated by the Mann–Whitney test.

**Figure 5.**
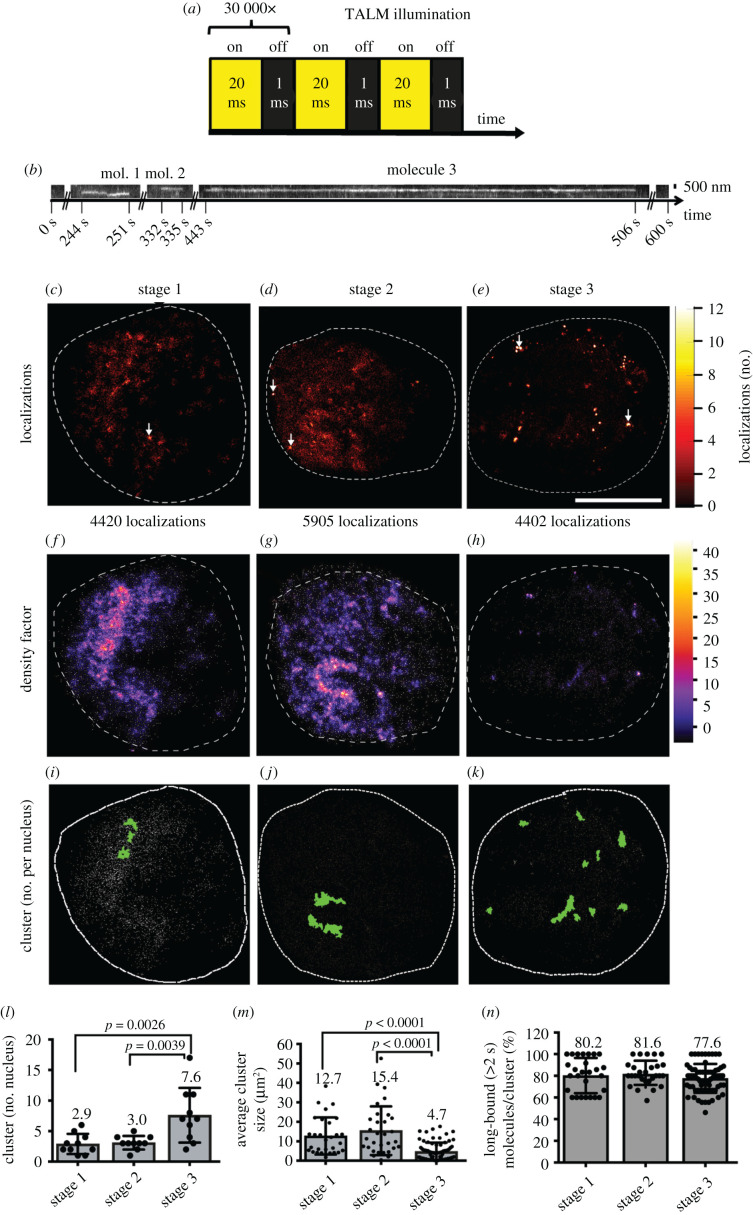
TALM analysis shows different cluster sizes and number of SRF long-bound molecules during neuronal differentiation. (*a*) TALM illumination scheme. In TALM, Halo-SRF molecules labelled with the photoactivatable dye JF-646-PA were illuminated for 20 ms with a 640 nm laser line. In the camera integration time, the JF-646-PA-dye was activated for 1 ms. One sample was imaged for 30 000 frames. (*b*) Representative kymograph of three molecules detected at an identical area during an entire TALM movie (600 s). *x*-axis indicates the time and *y*-axis the position. (*c–e*) Upscaled super-resolution image of the detection map of all Halo-SRF localizations which were linked to tracks longer than 1 s in the nucleus of stage 1 (*c*), stage 2 (*d*) and stage 3 (*e*) neuron. The lookup table indicated the number of Halo-SRF localizations per pixel of the upscaled image (pixel size is 26 nm). White arrows indicate pixels with the highest number of localizations. (*f–h*) Local density factor map of initial Halo-SRF localization positions for exemplary neurons from stage 1 (*f*), stage 2 (*g*), and stage 3 (*h*). The local density factor was calculated by the Voronoi tesselation and is indicated by the lookup table. (*i–k*) Visualized clusters (green area) of initial Halo-SRF localization positions (white dots) in a neuron at stage 1 (*i*), stage 2 (*j*), and stage 3 (*k*) together with overlaid initial positions of Halo-SRF molecules determined in the respective movie (grey symbols). (*l*) Average number of Halo-SRF localization clusters in stages 1, 2 and 3, determined by SR-Tesseler cluster analysis algorithm. Data were displayed as mean ± s.d. *n* = 9 cells for each stage. *p* values were calculated by Mann–Whitney test. (*m*) Computed average size of Halo-SRF clusters, data are presented as mean ±s.d. One circle depicts one cluster analysed. *n* = 26 clusters (stage 1); *n* = 28 clusters (stage 2); *n* = 63 clusters (stage 3). *p*-values were calculated by Multicomparison ANOVA test. (*n*) The percentage of long-bound (greater than 2 s) molecules per cluster is depicted for all three stages. One circle depicts one cluster analysed (for *n* numbers see (*m*)). Scale bars: (*b*) 500 nm; (*c–k*) 5 μm.

Neurons were prepared from the hippocampus of postnatal (P) J57BL/6 mice (P0–P2). We used 0.25% trypsin-EDTA (Gibco) to dissociate the hippocampal tissue. The tissue was then triturated in DMEM/10% horse serum (Gibco). The cells were then cultivated in NMEM/B27medium without phenol red (Gibco), supplied with 2% B27 supplements (Gibco), 0.6% glucose, 2 mM L-Glutamine (Gibco), 0.2% NaHCO_3_, 1 mM Na-Pyruvate (Gibco) and 5 µg ml^−1^ gentamicin (Gibco). We plated 250.000–400.000 cells per 35 mm cell culture dish with a 0.17 mm glass-bottom and a 500 µm grid (IBIDI). Depending on the experimental settings, neurons were either electroporated (differentiation experiment; figures 2–5; Amaxa Nucleofector, Lonza) with the pLV-TetO-Halo-mSRF plasmid immediately before plating or lentivirally transduced 2 h after seeding (BDNF stimulation; figure 1). Cells were cultured for up to 10 days at 37°C and 5% CO_2_. The electroporation was performed in accordance with the manufacturers cell line-specific protocol with 4 µg plasmid DNA. For the lentiviral transduction, the cells were cultured for 3 days at 37°C and 5% CO_2_ after adding the lentivirus dropwise in a 1 : 100 dilution. Then the cells were washed with NMEM/B27 for 2 consecutive days.

#### 2.2.2. NIH 3T3 cell culture

Stable NIH 3T3 cell lines expressing SNAP-MRTF, Halo-SRF and SNAP-MRTF or SNAP-Tag only were generated as before [5]. These cell lines were cultured with DMEM/GlutaMax (Gibco) containing 10% FCS (Gibco) and 1% penicillin/streptomycin according to standard cell culture procedures.

### 2.3. Instrumentation

The single-molecule live-cell imaging and the dSTROM measurements were performed with a custom build microscope described previously [32] with the ability to shift the excitation beam allowing for either epi-, HILO- and TIR-illumination. For the excitation of SiR, Janelia 646 and Alexa647, we used a 640 nm laser line (iBEAM-SMART-640-S, TOPTICA Photonics AG). A 532 nm laser line (Cobolt Samba, Cobolt AB) was used for the excitation of TMR and Alexa532. For photoactivation of Janelia JF646-PA, we used a 402 nm laser line (iBEAM-SMART-405-S, TOPTICA Photonics AG). The laser lines were coupled into a photonic crystal fibre (PCF) and were guided into a high-NA objective (Plan APO 100×, NA 1.45 Oil, Nikon). The fluorescence light was filtered and detected by two electron-multiplying charge-coupled device (EMCCD) cameras (iXon 897 Ultra, Andor Technology for the red channel; iXon 897, Andor Technology for the green channel). The set-up was controlled by a custom-written LabView application, and the cameras were controlled by the Andor Solis software.

### 2.4. Single-molecule tracking

#### 2.4.1. Primary hippocampal cells

Before imaging, cells were labelled with Janelia Fluor JF646 Halo-Tag (kindly provided by Dr Luke Laevis, Janelia Laboratories, Ashburn, USA). For the BDNF stimulation experiments 1 pM and for the differentiation experiment 10 pM (increased concentration to account for the lower transfection efficiency) of the JF646 Halo-Tag ligand (HTL) were used, respectively. After labelling for 15 min at 37°C and 5% CO_2_, we washed the cells 3 times for 10 min with NMEM/B27 medium, supplied with 5 µg ml^−1^ gentamicin. We used 5 div (days *in vitro*) cultured hippocampal neurons for cell stimulation experiments (figure 1) and added 10 ng ml^−1^ BDNF (Preprotech, 10 µg) to the medium before imaging. The cells were measured for a maximum of 80 min and a stage incubator (Okolab, Ottaviano, Italy) controlled the temperature to 37°C and the CO_2_ content to 5%. For differentiation analysis, we used neurons cultivated *in vitro* for 5 h (stage 1), 1 day (stage 2), 3 days (stage 3), 7 days (stage 4), respectively.

#### 2.4.2. Stable NIH 3T3 cell lines

To investigate MRTF-A dynamics, 50 pM SiR-SNAP ligand was used to label SNAP and SNAP-MRTF-A molecules. To investigate the SMT colocalization of SNAP-MRTF-A and Halo-SRF we used 30 pM SiR ligand to label SNAP-MRTF and 15 pM TMR-Halo ligand was used to label Halo-SRF molecules. Cells were incubated with the respective ligand(s) for 30 min at 37°C and 5% CO_2_ in both cases. After 3 times washing with PBS, the cells were starved for 24 h using DMEM/GlutaMax containing 0.05% FCS and 1% penicillin/streptomycin. For SMT, we used OptiMEM (Gibco) containing 0.05% or 10% FCS and 1% penicillin/streptomycin. The cells were measured for a maximum of 60 min at 37°C and 5% CO_2_.

### 2.5. Tracking and localization microscopy (TALM)

For TALM [33], Halo-mSRF expressing primary neurons were cultured for 5 h (stage 1), 1 day (stage 2), and 3 days (stage 3) *in vitro* and then labelled with 25 nM photoactivatable JF646-HTL dye for 15 min at 37°C and 5% CO_2_ (kindly provided by Dr Luke Laevis, Janelia laboratories, Ashburn, USA). After labelling the cells were washed 3 times for 5 min with NMEM/B27 supplied with 5 µg ml^−1^ gentamicin. For TALM we used a 640 nm laser and recorded movies of 30 000 frames with an exposure time of 20 ms and an excitation power of 2 mW. For activation, we used a 405 nm laser line with an exposure time of 1 ms and a power of 1 mW interleaved between the 20 ms illuminating frames. To analyse the TALM movies we used the TrackIt Matlab application with tracking parameters indicated in table 1 [34]. The positions of all detected Halo-SRF localizations which were linked to tracks were used to create a heat map (detection map without spurious detections) using the TrackIt Matlab application. The positions are determined to sub-pixel precision with a two-dimensional Gaussian fit and were then accumulated in a two-dimensional histogram. To achieve a super-resolution image, we upscaled the image by dividing the original bin size by a scaling factor of 5 (Pixel size: 26 nm). The pixel values indicated by the Matlab ‘hot’ LUT (figure 5*c–e*) correspond to the number of detections in each pixel of the upscaled image. To further evaluate whether the Halo-SRF dense regions result from repetitive Halo-SRF binding events or from extensively long Halo-SRF binding events we extracted the initial positions of all Halo-SRF tracks and used them for further cluster analysis by SR-Tesseler [35] and StormGraph (Scurll *et al*., bioRxiv; doi: https://doi.org/10.1101/515627). Both cluster analysis tools compare the distance of a molecule to all the neighbour molecules to a mean distance (*r*_0_) which would be measured, when every localization would be equi-distantly distributed to each other. The fold distance change is depicted as local density factor. For SMT and TALM analysis, we used the TrackIt Matlab application with tracking parameters are stated in table 1 [34].

**Table 1.** Experimental settings and parameters.

SMT method	construct/cell line	TrackIt parameter	value
ITM	halo-SRF in primary hippocampal neurons (figures 1 and 2)	threshold factor	3
		tracking radius	1.8
		min. track length	2
		gap frames	0
		min. track length before gap frame	0
time-lapse microscopy	halo-SRF in primary hippocampal neurons (figure 3)	threshold factor	1.2
		tracking radius	0.564 (continuous)
			1.187 (500 ms TL)
			1.853 (2 s TL)
		min. track length	7 (continuous)
			7 (500 ms TL)
			3 (2 s TL)
		gap frames	0
		min. track length before gap frame	0
TALM	halo-SRF in primary hippocampal neurons (figure 5)	threshold factor	3
		tracking radius	1.8
		min. track length	50
		gap frames	1
		min. track length before gap frame	3
ITM	SNAP, SNAP-MRTF in NIH3T3 cells (figure 6)	threshold factor	3
		tracking radius	1.8
		min. track length	2
		gap frames	0
		min. track length before gap frame	0
ITM	halo-SRF/SNAP-MRTF colocalization (figure 7)	threshold factor	2
		tracking radius	1.8
		min. track length	2
		gap frames	0
		min. track length before gap frame	0

### 2.6. Direct stochastic optical reconstruction microscopy (dSTORM)

NIH 3T3 cells stably expressing Halo-SRF and SNAP-MRTF-A were fixed with 4% PFA in PBS for 20 min. After three times 5 min washing with PBS, the cells were blocked with a 1% BSA and 0.1% Triton-X-100 containing PBS buffer for 2 h under gentle shaking. For antibody labelling, we used a rat anti-SRF serum antibody (kindly provided by A. Nordheim, Tübingen University, Germany) in a dilution of 1 : 200 and a rabbit anti-MRTF-A serum antibody (kindly provided by G. Posern, Halle University, Germany) in a dilution of 1 : 500 as primary antibodies The primary antibodies were incubated over night at 4°C under gentle shaking. A goat anti-rat Alexa Fluor 647 IgG (Invitrogen, A-21247) and a goat anti-rabbit Alexa Fluor 532 IgG (Invitrogen, A-11009) in a dilution of 1 : 1000 were used as secondary antibodies. The secondary antibodies were incubated for 1 h at room temperature under gentle shaking. The prepared samples were then covered with imaging buffer (PBS buffer with pH 7.5 containing 100 mM β-Mercaptoethylamine, 0.02 mg ml^−1^ catalase, 0.5 mg ml^−1^ glucose oxidase, and 200 mM glucose). The photo-switching of Alexa Fluor 647 and Alexa Fluor 532 was achieved by epi-illumination using a 640 nm (42 mW) or 532 nm (32 mW) laser line and the exposure time was 30 ms for both channels. The laser power was measured in front of the objective. The single-molecule localizations were corrected for chromatic aberration and reconstructed using the software SMAP [36]. The localization density (no. localizations per area) ratio between the nucleus and the cytosol was calculated for MRTF-A localizations for quantitative analysis by SMAP [36].

### 2.7. Image analysis and quantification

To determine the bound fraction and the residence time of Halo-SRF, we used an integration time (*τ*_int_) of 50 ms and a laser power of 2 mW (see figures 1–3). To determine the bound-fractions of Halo-SRF molecules, we used an illumination pattern called interlaced time-lapse microscopy [37]. Here, two consecutive frames with 50 ms integration time were followed by a dark-time of 2 s. Molecules surviving at least one dark-time time in an area of 0.24 µm^2^ were classified as long-bound molecules, whereas those detected in two consecutive illumination frames were classified as short-bound. The ratio between long-bound to all bound (long- and short-bound) events was calculated cell-wise (equation (2.2)) or molecule-wise (equation (2.3)), respectively. The JF646 labelled Halo-SRF was illuminated with the 640 nm laser line with a power of 2 mW. For measuring the Halo-SRF residence time, we performed time-lapse imaging by inserting dark-times between two consecutive illumination frames and varied between 0 and 2 s, to be able to correct for photo-bleaching. Tracking parameters were adapted for different time laps conditions are stated in table 1 and were chosen to achieve comparable photobleaching rates in hippocampal neurons in differentiation stage 1, 2 and 3. For further analysis, we used a fixed photobleaching rate of 0.0304 frame^−1^ which was determined by the average of these three bleaching rates. To obtain the dissociation rate spectrum, the survival time distribution of all bound molecule durations of continuous movies or the time-lapse datasets was calculated by TrackIt [34]. Then, the dissociation rate spectra were extracted in a global analysis of all time-lapse datasets. The resulted data were then fitted using a three-rate decay model including three dissociation rate constants. As an error estimation, we implemented a resampling of 80% of the data obtained by a superposition of 499 fit results [34]. Additionally, an ITM bound fraction was simulated from the results of the binding time measurements. As a starting point for the simulation we used the event spectrum of the three-exponential fit. To account for the fact, that short bound molecules are not detected during the dark-time in ITM, we multiplied the probability to detect an event with binding time $\tau_{\mathrm{bind}}$ to the event spectrum.

2.1 $$S_{ITM}(k)=S_{\mathrm{event}}(k)\frac{\tau_{\mathrm{bind}}}{\tau_{\mathrm{bind}}+\tau_{tl}}\;\;\mathrm{where}\;\;\tau_{\mathrm{bind}}=k^{-1}.$$

To simulate TFs in ITM measurements (figure 3*m*), we first drew a random number from the new probability distribution $S_{ITM}(k)$ to decide for a particular dissociation rate of the simulated TF. Next, we determined the number of frames the molecule survived by drawing a random number from an exponential probability-distribution consisting of the bleaching number and the dissociation rate of the TF. We then classified the TF as short- or long-bound respectively, according to the same rules that have been applied in the evaluation of the measured ITM-movies. We repeated this procedure 10 000 times and calculated the long-bound fraction from the counts of long- and short-bound molecules. To obtain the error of the simulated long-bound fraction, we used all 500 resampling runs of the three-exponential fit to simulate the bound fraction and calculated the standard deviation of the simulation results.

For the colocalization studies, TMR labelled Halo-SRF was excited with a 532 nm laser using 2 mW each, whereas SiR labelled SNAP-MRTF was excited with a 640 nm laser line and a power of 2 mW, respectively. All the stated laser power was measured in front of the objective. To determine the bound-fractions of colocalized Halo-SRF molecules and SNAP-MRTF-A molecules, we used ITM. To evaluate the fraction of molecules that bound longer than 2 s, we used an implemented TrackIt function to increase the ITM dark-time artificially during the analysis [34]. All tracking parameters set in TrackIt are shown in table 1. Molecules were classified as long-bound molecules when the molecules were detected to survive 2 or 3 dark time periods resulting in an increased dark-time of 4 s and 6 s, respectively. Molecules detected as bound molecules shorter than 2 or 3 dark time periods were counted as short-bound events in this instance. Furthermore, we calculated the long-bound fraction either cell-wise (equation (2.2)) or molecule-wise (equation (2.3)).

For the Halo-SRF/SNAP-MRTF-A colocalization, the distinct coordinates per frame of bound SRF and MRTF-A molecules were recorded and compared to each other using a self-written Ruby script (code provided in supplement). To reduce noise, molecules only detected in a single frame were not taken into account. MRTF-A and SRF molecules whose position was localized within 290 nm of each other were classified as colocalized. This distance was used to account for the chromatic aberration. The number of colocalized molecules was counted, and the fraction of colocalized molecules was calculated using

2.2 $$\begin{array}{rl} & {\mathrm{frac}}_{\mathrm{cell}\;\mathrm{wise}}=\\ & \frac{\text{no. long bound molecules}}{\left(\text{no. long bound molecules}+\text{ no. short bound molecules}\right)} \end{array}$$

and

2.3 $$\frac{\Sigma \;\text{no. long bound molecules}}{\Sigma \;\text{no. long bound molecules}+\Sigma \;\text{no. short bound molecules}}$$

### 2.8. Quantitative real-time PCR

We isolated total RNA with RNEasy Kit (Qiagen) according to the manufacturer's instructions. The reverse transcription was performed with 1 µg RNA using reverse transcriptase (Promega) and random hexamers. We performed quantitative real-time PCR (qPCR) with the Power SYBR green PCR master mix (Takara) on a Light Cycler 480II (Roche). Each sample was pipetted in doublets, and the threshold cycle (*C*_t_) values were calculated by the L480 II Software. The RNA expression levels were determined in relation to *Gapdh* RNA levels. Primer sequences are provided in the supplement.

## 3. Results

### 3.1. Neuronal activation with BDNF enhances the DNA-bound fraction of SRF

In order to analyse DNA binding dynamics of single SRF molecules in living neurons, we transiently transfected cultured mouse primary hippocampal neurons with a Halo-SRF plasmid and fluorescently labelled the expressed protein [5] using a bright fluorescent dye (JF646). Living neurons were then imaged using HILO illumination generating a thin light sheet of a few micrometers [2,38]. Here, molecules were selectively excited in a thin optical section, thereby increasing the signal-to-noise ratio (figure 1*b*). In the recorded movies single molecules were localized in each frame with sub-diffraction limit accuracy. Labelling was chosen to yield sparse signal, so that each bright fluorescent spot can be attributed to a single-molecule localization of SRF. Such overexpressed Halo-SRF molecules can potentially compete with endogenous SRF for DNA interaction and thereby perturbing precise parameter analysis. However, a recent study showed that this is not necessarily the case since for MeCP2 and also RNA-Polymerase II endogenous and overexpressed TFs behaved almost identical in SMT [11,39].

To determine the fraction of long-bound SRF molecules we employed interlaced time-lapse (ITM) microscopy as before [5,37]. Here, molecules were tracked over repeated cycles of 2 × 50 ms exposures interspersed by 2 s of dark-time (figure 1*d*). ITM allowed to differentiate between non-binding, short- and long-bound SRF subpopulations. We defined a single TF molecule as short-bound if it was only present in two successively illuminated frames, and as long-bound if it ‘survived’ in an area of 0.24 µm^2^ over at least one 2 s dark-time (figure 1*d*). During the recorded movies tens to hundreds of localizations were obtained from each cell and classified as long and short binding events (figure 1*e,f*).

First, we wanted to compare the fraction of long pauses for unstimulated as well as growth stimulated cells. Therefore, BDNF was applied to the growth medium for 80 min and data were presented as time bins of 20 min after stimulation (figure 1*g–i*). In unstimulated neurons, 30.4% of all SRF molecules were part of the long-bound TF fraction. This is in agreement with our previous result [5] using a different technical set-up and analysis (see Material and methods). During BDNF administration, the percentage of long-bound SRF molecules increased by approximately 30% to 39.1% within the first 20 min. Between 20–40 min of BDNF application, the long-bound SRF fraction slightly dropped by more than 10% only to rise again between 40–60 min of BDNF stimulation (figure 1*g*). In the last 20 min of BDNF application (60–80 min), the long-bound fraction of SRF molecules decreased again (figure 1*g*). Thus, while for all time-points the fraction of long bound molecules is higher, than for the unstimulated case, overall a wave-like pattern of SRF binding was observed. In order to analyse whether this wave-like pattern is modulated by an SRF-MRTF interaction we employed an SRF mutant protein (SRF *α*I helix) harbouring point mutations in the alpha helix, thereby impairing SRF interaction with MRTFs [31]. Neurons expressing a Halo-tagged version of this SRF *α*I helix mutant protein were left unstimulated or stimulated for 20 min with BDNF, followed by imaging with the ITM protocol (figure 1*g*; dark grey bars). By contrast to an enhanced fraction of DNA-bound WT SRF proteins (figure 1*g*, light grey bars), BDNF failed to enhance the fraction of long-bound SRF molecules if SRF-MRTF interaction was precluded by the presence of the SRF *α*I helix mutant protein (figure 1*g*). This suggests that interfering with an SRF-MRTF interaction prevents formation of an enhanced fraction of long SRF binding events to chromatin as already seen in fibroblasts [5].

While the observed changes during the stimulation are relatively small, statistical analysis proves their significance (see Material and methods), therefore also confirming the results of an earlier study [5]. The observed wave-like pattern can be linked to BDNF, since control neurons without BDNF, neither showed an initial rise of the long-bound fraction nor the described wave-like pattern (figure 1*h*). Moreover, BDNF stimulation increases the fraction of the long binding event, but not the overall binding on-rate, since the fraction of all-bound molecules to all molecules was not changed (figure 1*i*).

In summary, using a different technical and quantification regime we could confirm that the fraction of long-bound SRF molecules in primary hippocampal neurons was enhanced by growth factor stimulation and follows a wave-like pattern.

### 3.2. The long-bound SRF fraction is altered at several stages of neuronal polarization

Neurons undergoing differentiation pass through several stages characterized by initial adhesion (stage 1; figure 2*a,e*), growth of 4–5 neurites (stage 2; figure 2*b,f*), pronounced growth of the future axon (stage 3; figure 2*c,g*) and dendrite specification (stage 4; figure 2*d,h*) [1,40]. We therefore wanted to investigate the influence of these different stages on the DNA binding dynamics of SRF.

We employed our Halo-SRF construct to follow changes in the long-bound TF binding fraction along these four stages. To achieve Halo-SRF expression, electroporation was employed since viral infection takes too long to achieve expression in stage 1 (covering 5 h after plating). Once again, ITM illumination (figure 1*d*) was employed and 49 cells (stage 1), 98 cells (stage 2), 35 cells (stage 3) and 33 cells (stage 4) were analysed. SMT analysis of ITM movies allowed to determine the positions of short- (green) and long-bound (red) SRF binding events in the nucleus for each stage (figure 2*i–l*). When comparing many cells, no obvious pattern for the nuclear positioning of such long- or short-bound SRF events was discernable for each of the four stages and they appeared randomly dispersed all over the nucleoplasm (figure 2*i–l*; for a more refined analysis of TF clusters figure 5).

Stage 1 covers the initial adhesion of freshly dissected neurons to the laminin coated substrate. Here, the long-bound SRF fraction was highest with approximately 45% in ITM quantification with 2 s dark-time (figure 2*m*). In stages 2 and 3 the long-bound SRF fraction significantly dropped in both stages to roughly 35% (figure 2*m*). This percentage is in accordance with our previous results (figure 1 and [5]) where likewise stage 2 to 3 neurons were employed. In stage 4, typically reached after 7 d of culturing, neurons were more or less fully differentiated and engaged in several contacts with other neurons. Here, the SRF long-bound fraction was lowest for all stages with only about 31% (compared to 45% at stage 1) of all SRF molecules engaged in >2 s DNA binding (figure 2*m*). Since stage 1 neurons were analysed around 5 h after electroporation, this high percentage of long-bound SRF molecules might simply reflect an acute cell stress response. However, when the few stage 2 neurons which were present already at 5 h after plating were analysed, such a high long-bound fraction was not observed (electronic supplementary material, figure S2). This indicates a specific rise in longer SRF-DNA interactions during initial cell adhesion in stage 1.

We corroborated these findings by additionally quantifying long-bound events that passed two (4 s; figure 2*n*) or even three dark-times (6 s; figure 2*o*). Expectedly, the percentages of SRF molecules engaged in such extended DNA interaction dropped in all four stages for a 4 s dark-time (figure 2*m*) and even more pronounced for a 6 s dark-time (figure 2*n*). Nevertheless, as seen with a 2 s dark-time (figure 2*m*), in stage 1 the percentage of long-binding SRF molecules was always highest (figure 2*n*,*o*) and significantly reduced at later stages of differentiation. As before for the 2 s dark-time analysis, in stage 4 the long-bound fraction was the lowest also for 4 s and 6 s dark-time durations (figure 2*n,o*). When instead quantifying changes in all bound molecules (short and long) to the entire SRF population (regardless of bound or not bound) similar tendencies were observed, however without reaching statistical significance (figure 2*p*). However, it has to be kept in mind that the fraction of long-bound molecules is only approximately 1/3 (36.1%, 32.7%, 35% and 32.3% for stages 1–4, respectively; figure 2*p*) and the short-bound fraction comprises approximately 2/3 of all binding events (63.9%, 67.3%, 65% and 67.7% for stages 1–4, respectively; figure 2*p*). Therefore, when quantifying data with all-bound molecules, the short-bound population has a larger influence on the overall outcome and might outweigh the impact of the long-bound molecules. Thus, when looking at all-bound molecules, changes are typically less obvious in opposite to quantifications focusing on long-bound molecules only (figure 2*n,o*).

A previous SMT study showed that nuclear volume affects DNA binding of TFs [37]. Thus, we analysed whether neuronal differentiation along the stages was accompanied by changes in nuclear area (figure 2*q*). Indeed, stage 1 nuclei had significantly lower areas compared to stage 2 and 3 neurons whereas no difference to stage 4 neurons was observed (figure 2*q*) indicating that beyond a simple concentration effect there are other processes important for changing the binding duration of SRF.

Taken together, we observed significant variations in SRF binding during neuronal polarization with the largest SRF subpopulation of long DNA interaction during earliest rather than later stages of cell differentiation.

### 3.3. DNA residence times change during neuronal polarization

In the experiments described until now, we determined that the DNA-bound fraction consists of approximately one-third of all molecules in accordance with our previous report [5]. Those bound molecules typically segregate into two TF subpopulations, short- and long-bound molecules, as reported before [30]. Short binding (less than 1 s) molecules are binding unspecifically to chromatin for target search whereas longer binding molecules (greater than 1 s) are considered to bind to specific promoter sequences for active transcription [41]. In the next step, we quantified SRF residence times along the first three stages of neuronal differentiation for such fractions of chromatin-bound SRF molecules (figure 3).

To determine chromatin residence times, we used the recently developed genuine rate identification method (GRID) [42]. Therefore, we acquired continuous illumination movies (cont.) consisting of 200 frames of 50 ms laser exposure times to resolve short binding events (figure 3*a*). For longer binding events, time-lapse movies with 50 ms laser exposure times interspersed with varying dark-times (0.5 and 2 s) were recorded (figure 3*a*). Using GRID [42], acquisition of time-lapse movies with even higher dark times as done in an earlier study of SRF binding [5] was not necessary. In general, TFs ‘surviving’ longest dark times between two illuminations have highest residence times.

As with ITM (figure 2), primary neurons were electroporated with Halo-SRF and cultured for the first three differentiation stages (stage 1–3). For each stage, we recorded the time a bound-molecule was visible (figure 3*b*) and collected these times in survival time distributions (figure 3*c–e*). Exemplary kymographs of such representative long binding events for all three illumination regimes are shown in figure 3*b*. To account for cell movements, SRF molecules remaining within an area of up to 0.075 µm^2^ (cont.), 0.16 µm^2^ (500 ms) or 0.24 µm^2^ (2 s) were considered bound (figure 3*b*; see Materials and methods).

Previously, we used a three-rate decay model to describe residence times of SRF in the context of cell stimulation by growth factors [5]. Here, SRF-DNA interactions at a particular chromatin position belonged to one of three residence time regimes (short, intermediate and long residence time). However, as is the case for ITM, it has to be kept in mind that the same SRF molecule might switch to a different regime at any later timepoint not covered by the movie time span. Thus, it is best to describe the SRF binding dynamics by a spectrum of binding states rather than classes of molecules. The survival time distributions of Halo-SRF molecules obtained through all three stages of neuronal differentiation (figure 3*f–h*) did once again fit best to a three-component decay model (figure 3*c–e*). Thus, a model with three dissociation rate constants (k_off1_, k_off2_ and k_off3_; figure 3*f–h*) matched best, corresponding to three residence time regimes characterized by the respective average residence time (short, intermediate or long; figure 3*i–k*). The event spectrum of the corresponding dissociation rates revealed that events with the lowest dissociation rate (i.e. longest residence times) corresponded to 0.9% (stage 1; figure 3*i*), 1.3% (stage 2; figure 3*j*) and 0.6% (stage 3; figure 3*k*) of all events within a certain time window. Thus, for these longest-bound SRF fraction, changes in the range of 50% were seen between the three stages of neuronal polarization. The SRF subpopulation with intermediate k_off_ (intermediate SRF residence times) comprised 7.3% (stage 1), 6.1% (stage 2) or 4.9% (stage 3) of all binding events. Thus, also in this fraction of DNA bound SRF molecules the observed events change in the range of 20–30% along the three stages of neuronal differentiation (figure 3*i–k*). The fraction with the fastest dissociation rate (shortest bound fraction) typically covered the vast majority of binding events (around 92–95%) within a certain time window. Here, no major changes between differentiation stages were observed (figure 3*i–k*).

From the three dissociation rates, we calculated the respective average residence time resulting in three residence time regimes (long, intermediate, long) in all three stages (figure 3*l*). In stage 1, longest SRF DNA binding events (red, figure 3*l*) lasted on average 28 s and intermediate binding events (black, figure 3*l*) 2.5 s. By contrast, shortest binding events (green, figure 3*l*) were in the range of 0.2 s and this was unchanged for all three stages (figure 3*l*). DNA binding times of the long and intermediate fractions were decreased in stage 2 in line with a reduction of the longest-bound SRF fraction observed in ITM measurements (figure 2). In stage 3, the DNA residence times for the intermediate and longest bound SRF subpopulation were the highest with approx. 35 s and 3.2 s, respectively (figure 3*l*).

To compare the residence times and fractions of SRF in stages 1 to 3 obtained from the GRID analysis with the long-bound fraction obtained from the ITM measurement, we used the GRID results and simulated the fraction of long-bound molecules, using the same selection rules as in the ITM measurement (figure 3*m*). The simulation revealed 34% of all SRF molecules to be long-bound in stage 1, which reduced to 29% in stage 2 and 32% in stage 3, respectively (figure 3*m*). These values are in accordance to the long-bound fractions measured with ITM (figure 2*m*).

All in all, neuronal differentiation induces changes in residence times of SRF molecules that fall into three different binding fractions.

### 3.4. mRNA abundance of immediate early genes but not of actin cytoskeletal genes changes during neuronal differentiation

ITM (figure 2) and residence time (figure 3) measurements identified changes in DNA-binding by SRF when comparing stage 1 with stages 2/3. Therefore, we investigated whether SRF-regulated gene classes, IEG and cytoskeletal genes, followed a corresponding transcript abundance (figure 4). For this, qPCR analysis was performed in Halo-SRF electroporated neurons at stages 1–3 quantifying mRNA abundance of SRF-regulated IEGs and actin cytoskeletal genes in relation to the housekeeping gene *Gapdh* (figure 4).

*Srf* transcript levels comprising endogenous *Srf* as well as *Halo-Srf* transcripts were highest in stage 3 neurons most likely reflecting full *Srf-Halo* promoter activation at later compared to earlier time-points (figure 4*a*). However, at the protein level, Halo-SRF abundance was comparable between stages (electronic supplementary material, figure S3).

Next, several IEGs were analysed (figure 4*b–h*). Interestingly, all IEGs analysed had highest mRNA abundance in stage 1 and levels were reduced in both stages 2 and 3 without any obvious differences in the latter two stages (figure 4*b–h*). Thus, in stage 1, with a highest fraction and a long residence time of long-bound SRF molecules (figures 2 and 3), highest IEG mRNA levels were also observed. A similar pattern was observed for the axon guidance molecule *Sema3c* (figure 4*i*), a molecule involved in neurite outgrowth previously described to be regulated by SRF [22]. By contrast to the aforementioned genes, several SRF-regulated actin genes (figure 4*j–l*; data for *Acta1, Tpm2b* and *Myl9* not shown) were not consistently altered between the three stages.

### 3.5. TALM reveals alterations in cluster size and number of SRF long-bound molecules during neuronal differentiation

Previously, accumulations of long-bound TF molecules in clusters have been suggested to present transcriptional ‘hot-spots’ with high RNA Polymerase II activity [7,43,44]. In order to visualize Halo-SRF accumulations in clusters we performed TALM (tracking and localization microscopy; figure 5), which allows for high-resolution localization and diffusion analysis of single proteins in living cells [33,45,46].

In our TALM analysis, movies with a total duration of approx. 10 min consisting of 30 000 repeats of 20 ms laser activation followed by 1 ms photo-activation were acquired (figure 5*a*). In order to differentiate molecules likely involved in transcription events from those spuriously binding to DNA, we chose to display binding events only for molecules bound for greater than 1 s within an area of 0.24 µm^2^ (see exemplary kymographs, figure 5*b*). For each stage, greater than 9 neurons were analysed and the number of detected Halo-SRF localizations exceeding 1 s binding per cell was typically in the range of 4000–6000 (figure 5*c–e*). A heat-map showed Halo-SRF accumulations in all three stages (arrows in figure 5*c–e*) with brightest colours indicating up to 12 localizations per pixel (see scale in figure 5*c–e*). As ‘cluster’ we defined more than 5 initial position localizations in the same area. For each initial position image, a density factor calculation was performed and fold changes in the local density factor was provided by a heat-map (figure 5*f–k*). The local density factor signifies the local enrichment of SRF molecules in relation to an equidistant SRF distribution throughout the nucleoplasm exceeding that of the average density by a up to a factor of 40 (see Material and methods). Individual clusters were represented by green areas (figure 5*i–k*). Of note, such long-bound SRF-containing clusters were found in all parts of the nucleoplasm for all three stages and no obvious spatial map which would distinguish the three stages by cluster position was discernible. By contrast to cluster position, stage-specific differences in cluster number (figure 5*l*) and size (figure 5*m*) were observed. The number of clusters was more than two-fold higher in stage 3 compared to stages 1 and 2 (figure 5*l*). Conversely, the average cluster size was two- to three-fold reduced in stage 3 compared to the previous two differentiation stages (figure 5*m*). Additionally, we quantified the percentage of long-bound (greater than 2 s) SRF molecules per cluster which might be indicative of mediating active transcription (figure 5*n*). Here, approximately 80% off all Halo-SRF molecules in a cluster were bound for greater than 2 s irrespective of the differentiation stage (figure 5*n*).

In summary, cell differentiation in neurons was accompanied by changes in TF cluster size and number.

### 3.6. The fraction of long-bound molecules of the SRF co-factor MRTF-A is increased by serum stimulation

**Figure 6.**
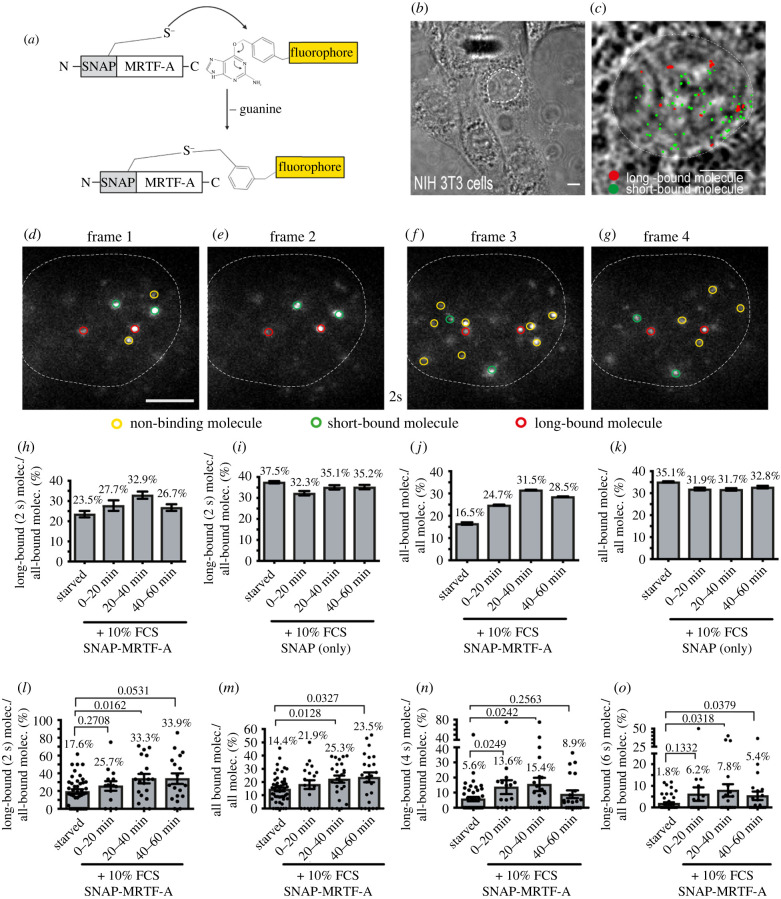
The fraction of long-bound MRTF-A molecules is increased by serum stimulation. (*a*) Schematic showing how SNAP-tag was N-terminally fused to the wild-type MRTF-A protein and was labelled by adding a selective fluorophore. (*b,c*) Bright field image of NIH 3T3 cells (*c*). Exemplary frames from an ITM movie of a NIH 3T3 cells expressing SNAP-MRTF, overlayed with the determined position from all bound molecules for the respective movie (short binding (green) and long-bound (red) molecules). Superimposed is the outline of the cell nucleus (white dashed line) determined from the respective bright field images. (*d–g*) Representative examples of molecules imaged over four frames and one dark-time interval of 2 s. Yellow circled fluorescent spots represent non-binding molecules. Two long-bound molecules were present in all four frames (red circles), whereas two short-bound molecules were present in frames 1 and 2 and two other short-bound molecules were present in frames 3 and 4 (green circles). (*h,i*) NIH 3T3 cells expressing SNAP-MRTF-A (*h*) or SNAP alone (*i*) were starved or stimulated with 10% FCS and the fraction of long-bound to all-bound molecules was computed. (*h*) *n* = 706 molecules, 26 cells for starved; *n* = 284 molecules, 21 cells for 0–20 mins FCS; *n* = 492 molecules, 25 cells for 20–40 mins FCS; *n* = 599 molecules, 20 cells for 40–60 mins FCS. (*i*) *n* = 2588 molecules, 26 cells for starved; *n* = 1846 molecules, 11 cells for 0–20 mins FCS; *n* = 2149 molecules, 15 cells for 20–40 mins FCS; *n* = 2172 molecules, 10 cells for 40–60 mins FCS. (*j,k*) The all bound fraction (short and long) was quantified for NIH 3T3 cells expressing SNAP-MRTF-A (*j*) or SNAP alone (*k*). (*j*) *n* = 4274 molecules for starved; *n* = 1336 molecules for 0–20 mins FCS; *n* = 1794 molecules for 20–40 mins FCS; *n* = 2056 molecules for 40–60 mins FCS. (*k*) *n* = 7745 molecules for starved; *n* = 6212 molecules for 0–20 mins FCS; *n* = 6773 molecules for 20–40 mins FCS; *n* = 6320 molecules for 40–60 mins FCS. Data in (*h*–*k*) were analysed molecule-wise and depict mean ± error. The error was calculated by a two-sample binomial test. (*l–o*) In cells expressing SNAP-MRTF-A the long-bound fraction (2 s; *l*) and all-bound (*m*) fraction was determined after FCS stimulation by cell-wise quantification. In order to account for binding times on the order of seconds also dark-times of 4 s (*n*) or 6 s (*o*) were analysed. Each dot reflects one cell (*n* = 26 cells starved; *n* = 21 cells 0–20 min FCS; *n* = 25 cells 20–40 min FCS; *n* = 20 cells 40–60 min FCS). Data in (*l–o*) were analysed cell-wise and depict mean ± s.d. *p*-values were calculated by the Mann–Whitney test. Scale bars: (*c,d–g*) 5 µm.

**Figure 7.**
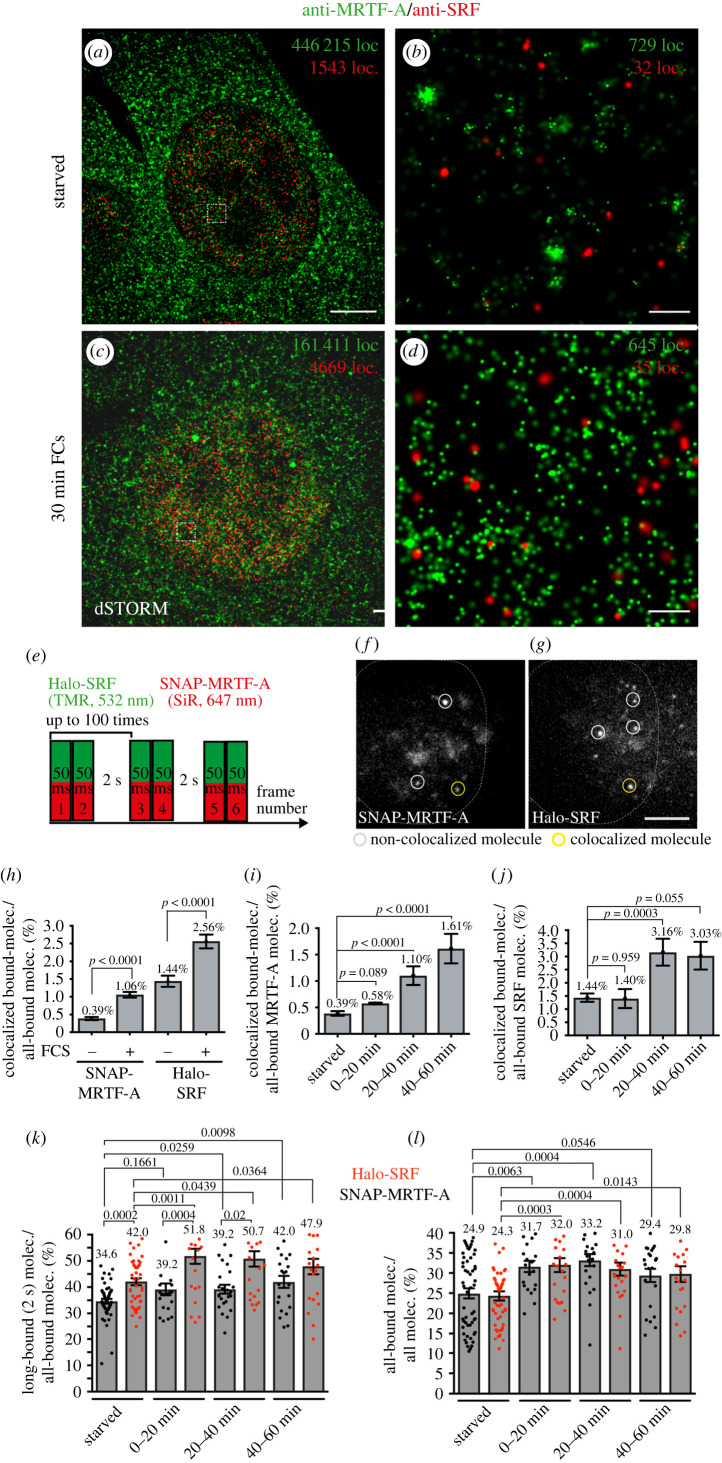
Enhanced SRF and MRTF-A colocalization in SMT experiments in living cells after stimulation. (*a–d*) Super-resolution optical microscopy (dSTORM) imaging of starved (*a,b*) or FCS-stimulated (*c,d*) NIH 3T3 cells simultaneously expressing Halo-SRF and SNAP-MRTF-A (*n* = 3 cells each condition). Cells were immuno-labelled with ant-MRTF-A (green) and anti-SRF (red) directed antibodies. Higher magnifications (*b,d*) of areas in boxes in (*a,c*) reveal clusters of localizations. (*e*) ITM illumination scheme with a simultaneous illumination of Halo-SRF stained with TMR (532 nm) and SNAP-MRTF-A stained with SiR (647 nm). The fluorophores were simultaneously illuminated for two consecutive frames of 50 ms, followed by a dark-time of 2 s. (*f–g*) Exemplary frame of an ITM movie taken from an NIH 3T3 cell expressing Halo-SRF and SNAP-MRTF-A. Images detecting SNAP-MRTF-A (*f*) and Halo-SRF (*g*) were recorded simultaneously using two separated camera channels. The yellow circle in both frames computed from an overlay of the two images indicates a SNAP-MRTF molecule colocalized with a Halo-SRF molecule. By contrast, the grey circles indicate SNAP-MRTF-A and Halo-SRF molecules non-colocalized (*h–j*) The bound fraction of SNAP-MRTF and Halo-SRF molecules was quantified for-colocalization in starved or stimulated NIH 3T3 cells. H: SNAP-MRTF-A (*n* = 21 191 molecules starved, *n* = 16 707 molecules stimulated) and Halo-SRF (*n* = 5707 molecules starved, *n* = 6680 molecules stimulated). MRTF-A molecules analysed for colocalization with SRF (*i*) were *n* = 21 191 (starved), *n* = 5276 (0–20 min FCS), 6885 molecules (20–40 min FCS) and *n* = 4546 molecules (40–60 min). Conversely, numbers for Halo-SRF molecules colocalizing with all-bound SNAP-MRTF-A molecules were *n* = 5707 molecules (starved), *n* = 2159 molecules (0–20 min FCS), *n* = 2374 molecules (20–40 min FCS) and *n* = 2147 molecules (40–60 min FCS). All data showed mean and *p* values and error were calculated using a two-sample binomial test. (*k–l*) In starved and stimulated NIH 3T3 cells co-expressing SNAP-MRTF-A and Halo-SRF, the long-bound (*k*) and all-bound fraction (*l*) of Halo-SRF (red) and SNAP-MRTF-A (black) molecules was determined cell-wise. Data in (*k,l*) were quantified cell-wise and were depicted as mean ± s.d. In (*k,l*) each dot represented one cell (*n* = 54 cells starved; *n* = 70 cells 0–20 min FCS; *n* = 25 cells 20–40 min FCs and *n* = 22 cells 40–60 min FCS). All *p*-values were calculated by the Mann–Whitney test. Scale bars: (*a,c*) 5 µm; (*b,d*) 200 nm (*f,g*) 5 µm.

Mouse mutagenesis of *Srf* and *Mrtfs* revealed similar phenotypes in differentiation of many cell types [21,25,47] indicative of a transcriptional complex established by SRF with its MRTF partner proteins. In order to investigate whether SRF and MRTF-A have shared and/or distinct properties, we performed SMT also for MRTF-A (figure 6). So far, MRTFs have not been analysed by SMT and in a first step, we addressed whether MRTF-A nuclear kinetics were influenced by cell stimulation in NIH 3T3 fibroblasts. By contrast to SRF, MRTF-A shuttles from the cytoplasm to the nucleus by cell stimulation, e.g. with serum [48,49]. To analyse MRTF-A in SMT we fused MRTF-A with a different SMT tag, the SNAP tag, resulting in SNAP-MRTF-A fusion protein (figure 6*a*). We used a tag orthogonal to the Halo tag to allow for later colocalization with Halo-SRF (figure 7). In a first step, we verified increased cytoplasm-to-nucleus SNAP-MRTF-A shuttling by serum in NIH 3T3 cells stably expressing SNAP-MRTF-A (electronic supplementary material, figure S4). As for SRF [5], we started SMT analysis on MRTF-A with an ITM illumination regime (figure 6*b–g*). Thus, short- and long-bound molecules were tracked over two consecutive 50 ms laser illumination interspersed by 2 s dark-times. As with SRF, the entire pool of short- and long-bound MRTF-A events was investigated and overlayed with the nuclear area (figure 6*b,c*). Single SNAP-MRTF-A molecules were found to either stay bound for at least one dark-time (long-bound, red in figure 6*d–g*), were only shortly bound (green in figure 6*d–g*) or detected in just a single frame (non-bound, yellow, figure 6*d–g*).

In starved fibroblasts, 23.5% of all bound molecules belonged to the long-bound MRTF-A fraction (surviving one dark-time of 2 s; figure 6*h*). After FCS incubation, the long-bound SNAP-MRTF-A fraction was increasing and after 20–40 min of FCS was 40% higher compared to baseline (32.9% versus 23.5%; figure 6*h*). An important control to be included was the SNAP-tag alone, since previously an unspecific DNA binding of the SNAP-tag was reported [50]. Of note, the abundance of SNAP-MRTF-A and SNAP-only mRNA was comparable as revealed by qPCR analysis (electronic supplementary material, figure S4). Indeed, in the absence of a TF conjugated to the SNAP-tag, this tag had a surprisingly high DNA affinity with a long-bound fraction of about 32%–37% depending on time-point (figure 6*i*). However, in contrast to SNAP-MRTF-A, the SNAP-tag alone did not show a specific trend upon FCS stimulation (figure 6*i*). The specificity of SNAP-MRTF-A responses is further corroborated by looking at the ratio of all bound molecules (long and short) over all molecules (figure 6*j*). Here, FCS stimulation almost doubled the percentage of SNAP-MRTF-A molecule engaged in either short or long binding in relation to all molecules regardless of being bound or unbound (starved: 16.5% versus 40 min FCS: 31.5%; figure 6*j*). Thus, MRTF-A-DNA interaction was strongly enhanced by serum stimulation (figure 6*j*). By contrast, the SNAP-tag alone responded to FCS stimulation with only minor changes in the range of 5% (figure 6*k*). Thus, although the SNAP-tag *per se* has a rather high baseline DNA affinity, once it is conjugated to MRTF-A it provides a useful tool for the analysis of TF properties.

Above, data were analysed molecule-wise irrespective of which cell molecules were present (see materials and methods). In our previous work, we provided cell-wise analysis of TF parameters with each cell rather than each molecule representing biological replicates [5]. Thus, to facilitate comparison to this previous data and also analysing robustness of data when comparing different cells with each other, we also included cell-wise data analysis (in figure 6*l–o* and electronic supplementary material, figure S5 each dot represents one cell). Cell-based quantification revealed an almost two-fold increase in either long-bound (figure 6*l*) or all-bound (figure 6*m*) MRTF-A molecules at a 2 s dark-time interval similar to molecule-wise analysis (figure 6*h,j*). Thus, cell- and molecule-based analysis deliver almost identical results. As before (figure 2), we also tested the effect of increased dark-times using either 4 s (figure 6*n*) or 6 s (figure 6*o*), thereby focusing on fractions of longest DNA-bound molecules. Here, FCS application at the 20–40 min time-frame enhanced the Halo-MRTF-A fraction almost three-fold (figure 6*p*) or in case of a 6 s dark-time even more than four-fold compared to the starved condition. As above, SNAP alone had a higher long-bound or all bound fraction compared to SNAP-MRTF-A which however was not obviously influenced by FCS (electronic supplementary material, figure S5).

In summary, the SRF cofactor MRTF-A showed a constant and robust increase in long-binding DNA interaction upon serum activation.

### 3.7. Enhanced SRF and MRTF-A colocalization after cell stimulation

Besides widely overlapping target genes and mutant phenotypes [51], SRF and MRTF-A were considered as partner TFs for instance by protein co-immunoprecipitation studies [52]. In order to analyse SRF and MRTF-A colocalization with super-resolution microscopy we performed dSTORM in fixed cells (figure 7*a–d*). Furthermore, we investigated a putative colocalization of SRF and MRTF-A with SMT in living cells which so far to the best of our knowledge was not accomplished for SRF and MRTF-A or any other two TFs (figure 7*e–j*).

In the first set of experiments, we employed dSTORM in fixed NIH3T3 cells stably expressing both Halo-SRF and SNAP-MRTF-A that were starved or stimulated with FCS (figure 7*a–d*; electronic supplementary material, figure S6). Cells (*n* = 3) were stained with antibodies directed against MRTF-A and SRF thereby labelling both endogenous and overexpressed SRF and MRTF-A proteins. As seen in fluorescence immunocytochemistry (electronic supplementary material, figure S4), MRTF-A was predominantly cytoplasmatically localized in starved cells (green in figure 7*a,b*; localization density nucleus/cytosol: 0.6) and shuttled to the nucleus upon FCS stimulation (figure 7*c,d*; electronic supplementary material, figure S6; localization density nucleus/cytosol: 1.5). By contrast to MRTF-A, SRF was localized constitutively nuclear irrespective of FCS application (red, figure 7*a–d*), in line with previous findings [5]. In the nucleus, both SRF as MRTF-A appears not homogeneously distributed but clustered (figure 7*b* and *d*).

In the next step we established simultaneous SMT analysis of two proteins in living cells (figure 7*i–n*) using NIH 3T3 cells stably expressing Halo-SRF (labelled with TMR) and SNAP-MRTF-A (labelled with SiR). In a modified ITM illumination scheme (figure 7*e*), two lasers simultaneously captured Halo-SRF and SNAP-MRTF-A in two consecutive frames with 50 ms exposure times each followed by 2 s dark-time, as before (figure 1). In those movies (*N* = 54 cells, unstimulated; *N* = 70, stimulated) we quantified SRF and MRTF-A localizations. To quantify only colocalizations of bound SRF and MRTF-A molecules, we detected events, where both molecules remained at the same area over 100 ms (2 frames) or longer, thus depicting short- or long-bound molecules (see Methods). An example of a spatio-temporal colocalization of SNAP-MRTF-A and Halo-SRF is provided in figure 7*f*,*g* (yellow circle). On this basis we quantified whether the number of Halo-SRF and SNAP-MRTF-A colocalization events would change upon stimulating starved fibroblasts with 10% FCS. This analysis was done for the entire 1 h FCS stimulation (figure 7*h*) and for time windows of 20 min after stimulation (figure 7*i,j*). Regardless of the time window, the overall percentage of colocalizing molecules was low and never exceeded 3% of all bound molecules (figure 7*h–j*). Nevertheless, we consistently observed that the number of bound Halo-SRF molecules colocalizing with SNAP-MRTF-A (figure 7*h,i*) and vice versa (figure 7*h,j*) was always increased upon cell stimulation with FCS. When plotting data for individual 20 min time bins we observed that highest colocalization percentages were achieved at the 40–60 min time interval after stimulation (figure 7*i*,*j*). Above, we only analysed SRF and MRTF-A colocalizing molecules bound for 100 ms or longer. Finally, we also measured the percentage of long-bound SRF molecules (bound longer than greater than 2 s, greater than 4 s or greater than 6 s) colocalizing with MRTF-A (electronic supplementary material, figure S7). Once again as observed above (figure 7*h,j*) also the percentage of long-binding SRF molecules colocalizing with MRTF-A was elevated by stimulation (electronic supplementary material, figure S7). These findings indicate more SRF-MRTF-A interaction upon cell stimulation which might result in formation of functional SRF/MRTF-A complexes involved in target gene regulation.

In previous experiments we analysed SMT parameters individually for either SRF (figures 1–3; [5]) or MRTF-A (figure 6) with different cell types and experimental set-ups thereby complicating direct comparison. Since we established an ITM protocol for simultaneous Halo-SRF and SNAP-MRTF-A imaging (figure 7*e–j*) we now can directly compare parameters in the same setting (figure 7*k,l*). In agreement with our previous results (figures 1–3 and 6; [5]) we observed for Halo-SRF and SNAP-MRTF-A an enhanced long-bound (figure 7*k*) and all bound (figure 7*l*) fraction after stimulation. For the long-bound fraction, the percentage of SRF molecules was approximately 10% higher compared to MRTF-A in starved and stimulated cells (figure 7*k*) and the all-bound fraction increased by about 25% (figure 7*l*). Overall, the long-bound fractions of both SRF and MRTF-A in this co-expression experiment were approximately 10% higher (figure 7*k*) compared to individual analysis (figures 1 and 6; [5]). This might reflect a synergistic effect for DNA interaction when SRF and MRTF-A were co-expressed but also differences in experimental set-up (i.e. labelling of proteins) might account for this.

Overall, these results showed that both SRF and MRTF-A follow a similar pattern after cell stimulation by increased engagement in DNA-bound fractions.

## 4. Discussion

### 4.1. Cell differentiation changes DNA-bound fractions, TF residence times and TF clusters

In this study changes in single-molecule TF kinetics during neuronal differentiation were uncovered. In a previous study, we observed in unstimulated stage 2–4 neurons a long bound-fraction of approximately 27% [5] fitting well with the 30–35% obtained in this study (figures 1 and 2). Also, residence time measurements for stage 2–3 neurons revealed that SRF subpopulations fall into three residence time regimes with comparable DNA residence times in the previous [5] and current study (short: 0.2 s, intermediate: 2.0 s, long: 15.7 s; figure 3). Overall, this underscores robustness of parameters identified despite technically different set-ups employed (e.g. dyes, microscope set-up, transfection protocols).

**Figure 8.**
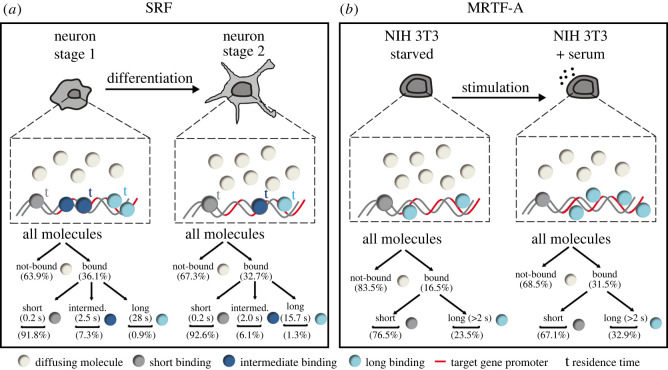
Summary scheme. (*a*) (top) Scheme depicting the changes in nuclear SRF kinetics during neuronal differentiation comparing stage 1 and stage 2 neurons. The DNA residence time (t) of long-bound (light blue) and intermediate-bound (dark blue) molecules were increased in stage 1 compared to stage 2. Higher residence times are indicated by larger letters in bold. In addition, the fraction of long- and intermediate bound SRF molecules (indicated by circle numbers) was also elevated in stage 1 compared to stage 2. (bottom) Percentages of bound-fractions and residence times were summarized for comparing stage 1 with stage 2. (*b*) Scheme depicting the findings for SMT experiments with SNAP-MRTF-A in starved and stimulated NIH 3T3 cells. In ITM experiments, serum stimulation enhanced the fraction of all-bound and long-bound MRTF-A molecules. No definitive binding times were calculated for SNAP-MRTF-A.

The most striking novel observation of this work was that during neuronal differentiation, long-bound fractions and DNA residence times were strongly increased during initial cell adhesion (stage 1; summarized in figure 8*a*). In stage 1, the SRF long-bound fraction (45%; figure 2) and residence times (intermediate: 2.5 s and long: 28 s; figure 3) were overall the highest compared to the later differentiation stages, particularly stage 2. Thus, it appears that during initial cell adhesion TF interaction with DNA was highest and decreased along cell maturation in later stages 2–4 (figure 8*a*). What might be explanations for an initially higher number and longer SRF interaction with DNA during cell differentiation? During stage 1, cells might be more stressed, however, enhanced cell stress did not obviously contribute to elevated SRF DNA interactions (electronic supplementary material, figure S2). As cells undergo differentiation their volume and nucleus size changes. Previous reports demonstrated a correlation between cell/nucleus size and gene transcription [37,53]. For instance, in zebrafish decreased nuclear volume correlated with increased DNA binding of TFs [37]. Likewise in the neurons investigated here, nucleus size was different between stages 1–3 (figure 2) and—as seen in zebrafish—the lowest nucleus area (stage 1) correlated with the highest SRF DNA association. Besides nucleus size, cell adhesion induces so-called adhesion-transcription coupling where nuclear lamina proteins such as lamins, emerin and the LINC complex (Linker of Nucleoskeleton and Cytoskeleton) translate changes in cell adhesion to gene transcription [54,55]. Interestingly, it is known in general that those molecules [56–58], cell spreading and cell-cell contact [59–62] also target MRTF-A nuclear shuttling and SRF/MRTF-A mediated gene transcription. Thus, it is conceivable that such enhanced adhesion-transcription coupling during initial neuron adhesion in stage 1 increases SRF/MRTF-A activity. Notably, in this regard, we were surprised to observe highest IEG (rather than cytoskeletal gene) mRNA abundance in stage 1 neurons (figure 4). Moreover, previous reports also showed induction of IEG transcription during cell adhesion in comparison to other differentiation states [63,64].

One exception to generally decreased TF kinetics in neuronal differentiation was for one parameter, i.e. the SRF DNA residence time in stage 3 neurons. Here, highest residence times were measured for the intermediate- and long-binding SRF fraction compared to stage 1 and 2 (figure 3). Since SRF molecules switch between these subpopulations, the SRF molecule numbers in those fractions can also vary (figure 3*i–k*). Indeed, we observed that in stage 3 the amplitude of the intermediate and long-bound fraction (4.9% and 0.6%, respectively) was about 30% smaller compared to stage 1 (7.3% and 0.9%; figure 3*i–k,m*). Thus, individual SRF molecules in stage 3 might associate longer with DNA but the percentage of molecules in this fraction was decreased compared to stage 1. In summary, our data suggest that the overall SRF activity in a given fraction might be adjusted by a feedback mechanism acting on (i) the DNA interaction time but (ii) also the molecule number joining such a subpopulation.

A third parameter quantified in cell differentiation besides TF bound-fraction (figure 2) and residence time (figure 3) was accumulation of SRF molecules in clusters by TALM (figure 5). Others have already used TALM protocols for membrane and mitochondrial proteins [33,45,46] as well as nuclear proteins [65,66]. In the nucleus, previous studies argued that such TF aggregates or clusters might indicate presence of so-called transcriptional hot-spots or hubs where specific gene clusters are predominantly transcribed [7,43]. In our study, formal proof of such activation hot-spots by showing e.g. colocalization with activated RNA Polymerase II is missing. In our experiments, we find that the vast majority of the SRF molecules localized in these clusters showed long-binding (greater than 2 s; figure 5*n*) which provides further evidence that SRF molecules are engaged in active transcription. Although speculative, such clusters might allow repeated binding of SRF to target gene promoters and represent transcriptional foci where specific SRF-mediated gene expression programs such as IEG transcription are locally and spatially concentrated within the nucleus. Future studies could provide further evidence to this hypothesis for example by determining the amount of transcriptionally activated Polymerase localized to these clusters.

Our TALM data showed that size (approx. 5–30 µm^2^) and number (2–10/nucleus) of such SRF clusters changed during neuronal differentiation (figure 5). Our hypothesis is that these SRF clusters depict hot-spots for differentiation stage-specific gene transcription programs. Previous studies in yeast uncovered that cell stimulation but also cell growth and maturation enhanced the TF cluster number [65,66]. The latter result would be congruent with our finding in neurons where likewise the highest SRF cluster numbers were observed in the most mature stage (stage 3; figure 5).

### 4.2. Cell stimulation enhances fractions of long-bound MRTF-A molecules and SRF colocalization

SRF and its MRTF cofactor form an important transcriptional unit involved in cell differentiation of many cell types [17–20]. So far, MRTFs or the SRF-MRTF interaction was not analysed with SMT. Herein we performed MRTF-A SMT (figure 6) but also a SMT analysis in cells co-expressing both tagged SRF and MRTF-A proteins (figure 7). Overall, all experiments showed that SRF and MRTF-A respond very similar towards cell stimulation by increasing the long-bound DNA fraction (figures 6 and 7; summarized in figure 8*b*) as also seen before [5]. Our data on the single-molecule level are congruent with MRTF-A chromatin immunoprecipitation (ChIP) experiments. Here, enhanced MRTF-A occupancy with DNA of target gene promoters was a result of TPA cell stimulation [67].

Of note, the absolute numbers for the fractions of long-bound and all-bound SRF or MRTF-A bound molecules were higher during co-expression (figure 7) as compared to single expression [5]. This might reflect synergistic properties of SRF and MRTF-A interaction. So far, SRF-MRTF interaction was mainly supported by shared phenotypes of mouse mutants, co-immunoprecipitation or overlapping gene programs [17,51,52]. Herein, a SMT colocalization study of TFs in living cells was performed. This analysis showed a two-to four-fold enhanced colocalization of DNA-bound SRF and MRTF-A after cell stimulation (figure 7). Thus, also SMT shows enhanced SRF-MRTF-A colocalization after stimulation which might be indicative of enhanced SRF/MRTF-A complex formation to drive gene transcription of serum-induced genes. Overall, the frequency of colocalization was low (0.4–3%), however, this number is low for technical reasons since only a small fraction of all molecules was labelled to ensure distinct single-molecule localizations. SRF colocalization events were highest at longest stimulation times (i.e. 40–60 min, figure 7*i,j*). This SRF/MRTF-A colocalization is rather late compared to the peak of some of the rapidly mediated SRF dependent gene expression (e.g. of IEGs) occurring often already between 10 and 30 min after stimulation and diminishing thereafter. Thus, although not experimentally addressed in this study, SRF/MRTF-A complexes might not only be involved in transcriptional activation but could also be involved in transcriptional repression. Indeed, mechanisms of transcriptional repression have been described for both, SRF and MRTF-A [68–70].

Taken together independent experimental set-ups, cell types (neurons, fibroblasts) and stimuli (serum, growth factor) show that both SRF and MRTF-A respond to cell stimulation with enhanced DNA occupancy.

## Data Availability

Additional data are provided in electronic supplementary material [71].
